# Entering the World of Sounds: An Autobiographical Case Report on Ethical Issues Surrounding Cochlear Implants

**DOI:** 10.7759/cureus.25320

**Published:** 2022-05-25

**Authors:** Konstantina Melissourgou, Giorgos Sideris, Thomas Nikolopoulos

**Affiliations:** 1 Otolaryngology, 2nd ENT Department, Attikon University Hospital, National and Kapodistrian University of Athens, Athens, GRC

**Keywords:** ethics, cochlear implant, pediatric, children, deafness

## Abstract

Cochlear implants are considered a viable therapeutic option for deaf children and adults who meet the implant criteria. They enable the hearing impaired to deal with the effects of deafness and participate academically, socially, and vocationally with their family and peers, aiding them to live a life with fewer limitations in the world of spoken languages. Most importantly, cochlear implants counteract the impact of profound deafness in early childhood, in which the ability to develop spoken language is severely restricted. For these children, cochlear implants represent the only means of hearing speech, resulting in the development of meaningful oral communication abilities. Over the past decades, there have been intense debates about communication philosophies for deaf or hard-of-hearing children, the nature and concept of deafness, the value and diversity of the deaf culture, the purpose of deaf education, and the best education systems for deaf or hard-of-hearing children. There are two broad camps in the debate over cochlear implantation: those who embrace the technique and those who oppose it. In this paper, we describe my own journey from total deafness to the beginning of a dream career as an otorhinolaryngology resident. We also engage in a discussion about the ethical concerns associated with cochlear implantation.

## Introduction

While the first known attempt to use electricity to enable hearing goes back to 1790, when the researcher Alessandro Volta placed metal rods in his own ears and connected them to a 50-volt circuit, it was actually in the 1980s that cochlear implants received the stamp of Food and Drug Administration (FDA) approval for implantation in adults, thereby ushering in wide-ranging advancements in speech processors and implant technology, particularly the miniaturization of the speech processor [1]. Cochlear implants are the latest technological achievement in dealing with the problem of deafness and represent the electronic equivalent of the cochlea (mainly the aesthetic hair cells of the Corti organ). It is an artificial sensory organ that bypasses the hearing system and directly stimulates the ends of the auditory nerve [2]. The placement of a cochlear implant is suggested for the recovery of patients with bilateral severe hearing loss or deafness who cannot benefit from hearing aids. For children to be eligible to receive cochlear implants, they must demonstrate a severe degree of hearing loss or deafness, absence of damage to the auditory pathway of central etiology or lack of auditory nerve, failure in the use of hearing aids for at least three months, should be at least 10 months old and able to undergo general anesthesia, should have family support, motives, and appropriate expectations, and support through education and recovery for the development of spoken language, speech, and hearing [3].

There are two major schools of thought with respect to cochlear implantation, and these comprise the supporters and opponents of the technology.

## Case presentation

I had never faced any hearing issues prior to my deafness and had no significant medical history. At the age of eight, I began experiencing severe headaches. It started in the early afternoon of March 28, 2001. As time passed, the symptoms progressed to dizziness and retching. I remember walking down the hall with difficulty one day and approaching my mother in tears, informing her that I was ill. My mother called our family doctor and described my symptoms to him. He then advised that if I developed a fever, I should seek medical attention immediately and that I was most likely suffering from a viral infection.

That same night, my fever spiked to 37.5 °C, which was quickly reduced with simple antipyretic medications, while I kept a large bowl nearby in case I needed to vomit. The following day, I felt better, but my head felt heavier, and I had no appetite. During the day, the sunlight bothered me, and I expressed my discomfort and confusion through my words and actions. My mother called an ambulance, and during the drive, I experienced epileptic seizures.

I was diagnosed with meningoencephalitis caused by pneumococcus based on a thorough lab examination, scanning, and spinal tap. I was moved to isolation and, following a dramatic deterioration of my condition, admitted to the pediatric ICU. The doctors had prepared my mother for the worst-case scenario, as my health was deteriorating and every moment was critical both for my survival and to arrest the disease's progression.

I was in the ICU for two weeks in total, and following that, I was eating via nasogastric tube, had poor sphincter control, and was completely unable to walk. The complication of deafness became apparent two weeks later when my family was unable to communicate with me effectively due to my inability to respond appropriately to their questions. My hearing loss primarily affected me psychologically, by causing melancholy, which resulted in social isolation. When I gradually regained my mental capacity, the doctors began looking for a cure for my bilateral neurosensory deafness.

Cochlear implantation was proposed following a failed attempt with hearing aids to improve my hearing and speech. Due to my recent meningitis history, I was required to undergo a regular temporal bone MRI to monitor for possible cochlea non-ossification. Finally, on May 12, 2002, I underwent cochlear implantation in the right ear.

Following successful implantation, I attended months of intensive speech therapy classes to maximize the implant's capabilities in conjunction with speech therapy intervention. Through speech therapy, I improved my conceptual ability in speech and hearing without the use of lip-reading. Additionally, I engaged in extensive play therapy and sessions with an expert psychologist, which aided me in comprehending the world around me in these new circumstances.

I continued my education in a public school without receiving any additional assistance or encountering significant difficulties. I first became acquainted with sign language when I was 17 years old, approximately nine years after I was diagnosed with hearing loss, and was introduced to the deaf community. Initially, I encountered the deaf community's disbelief and suspicion, followed by judgment, as I did not know sign language and was a cochlear implant user. Eventually, in order to communicate with deaf people, I learned sign language.

## Discussion

Hundreds of thousands of people of all ages are believed to benefit from cochlear implants, which enable them to hear properly and thereby fully engage with the complexities of their environment and contribute actively and creatively to society. According to the author, cochlear implant technology opens doors previously closed to the deaf, allowing the hearing-impaired to overcome the effects of deafness and participate academically, socially, and professionally with their family and peers, enabling people to live a life with fewer limitations, and maximizing their capacities and opportunities [4]. Most importantly, cochlear implants counteract the impact of profound deafness in early childhood, in which the ability to develop spoken language is severely restricted. For these children, cochlear implants represent the only means of gaining the ability to hear speech, resulting in the development of meaningful oral communication abilities.

Many studies have shown that the majority of hearing-impaired children find reading difficult, leading to significantly lower levels of reading attainment than their hearing peers throughout their school years. Moreover, this gap between deaf children and their hearing peers tends to widen with age [5]. However, there is considerable variability among cochlear implant users’ reading competence as revealed by linguistic competence, speech production skills, and phonological abilities. The age at which a child receives the implants is critical for the development of reading abilities. Archbold et al. found that reading abilities following cochlear implantation were associated with chronological age at five and seven years post-implantation in children who received implants before the age of 42 months, but not in children implanted after 42 months. These findings demonstrate that cochlear implantation has a beneficial effect on the development of reading skills in deaf children by promoting literacy [6].

It is critical to emphasize, however, that supporters of this procedure feel that cochlear implantation should remain a personal choice and should not be made mandatory for all deaf people. Additionally, when patients are minors, the decision to implant a cochlear implant is made by the parents, who have been informed about the procedure and opt to incorporate the child's "hearing culture" [7-8]. Moreover, new research has revealed significant discrepancies in access to cochlear implantation treatments in developed countries, highlighting the need for public policy measures. Implantation rates vary significantly between industrialized and developing countries, as well as among minority groups, indicating significant disparities in potential implantees based on the presence of another impairment and socioeconomic levels [9].

Despite the benefits of cochlear implant technology, there have been persistent debates over the last several decades about communication philosophies for deaf or hard-of-hearing children, the nature and concept of deafness, the value and diversity of deaf culture, the purpose of deaf education, and the best education system for deaf or hard-of-hearing children. These debates revolve around discrepancies between theory and practice; ethical concerns about cochlear implant surgery at an early stage, both medically and socially; informed consent; the parental decision-making process; and the annihilation of residual hearing [10].

Primarily, the sociocultural approach establishes that the deaf community does not view itself as a medically curable disability group. The community has a tendency to regard deafness as an aspect of difference rather than a hearing impairment, portraying it as a natural state. Additionally, the deaf community's principal form of communication, American Sign Language (ASL), which is a language in and of itself with its own grammatical elements and styles of expression, is viewed as their "native language". Also, it has been observed that deafness inspires and sustains a unique language and culture that enables meaningful engagement in all facets of life and society [9-12].

Additionally, members of the deaf activist community believe that trending YouTube videos of cochlear implant activation sensationalize and romanticize cochlear implants, offering a skewed perspective by way of sugarcoating the shock and anguish experienced by many recipients [13]. Furthermore, the community believes that parents who do not have hearing impairments should refrain from interfering with their deaf children's decision-making regarding cochlear implants, as these are viewed as a tool for eradicating sign language and the deaf community's sense of identity as a whole. Additionally, it is advised that informed consent for cochlear implantation should incorporate deaf individuals' testimonies [11].

Crouch, in a study, further argues that opting out of cochlear implantation for one's child does not doom them to a world of meaningless quiet, but rather exposes them to the deaf community, which is historically, linguistically, and morally a rich and rewarding experience [14]. Research conducted in Tokyo, Japan on the decision-making process of parents prior to their child receiving a cochlear implant indicated that guardians face difficulties in making decisions and require information and support from a variety of professionals, deaf adults, and children with implants themselves, while some of them give their children the option of having the implant removed if they so desire [7]. Despite the difficulties associated with decision-making, parental expectations before implantation are high (81%) in the areas of communication and development of spoken language, but expectations with regard to their children gaining the ability to listen to speech without lip-reading are much lower (35%) [8].

**Figure 1:**
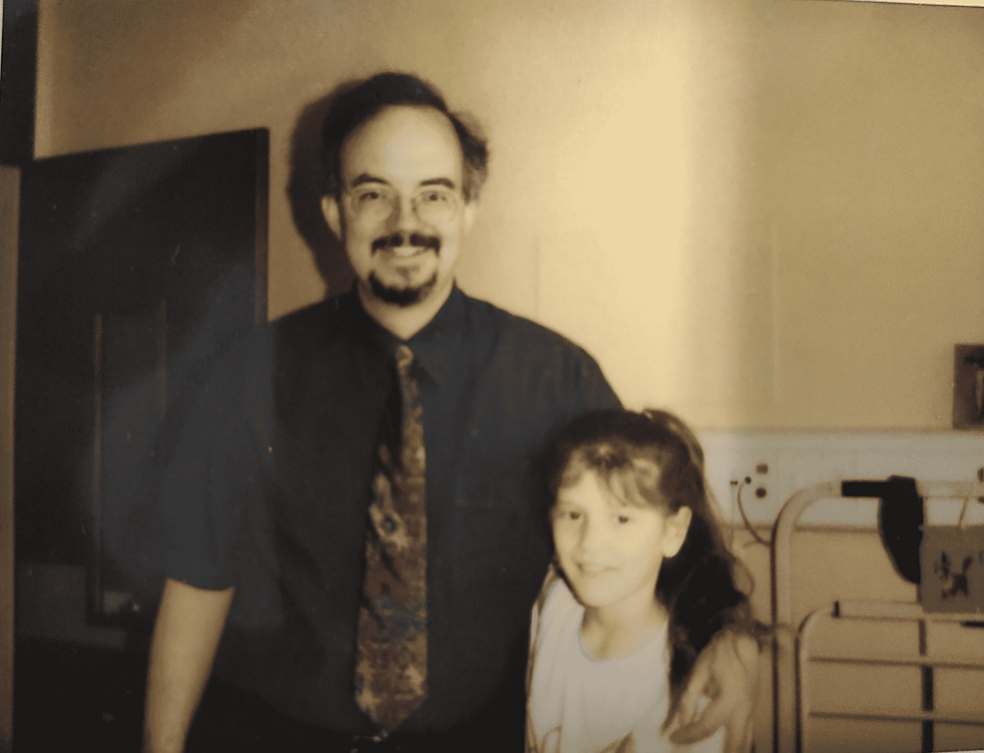
Me and my surgeon

**Figure 2:**
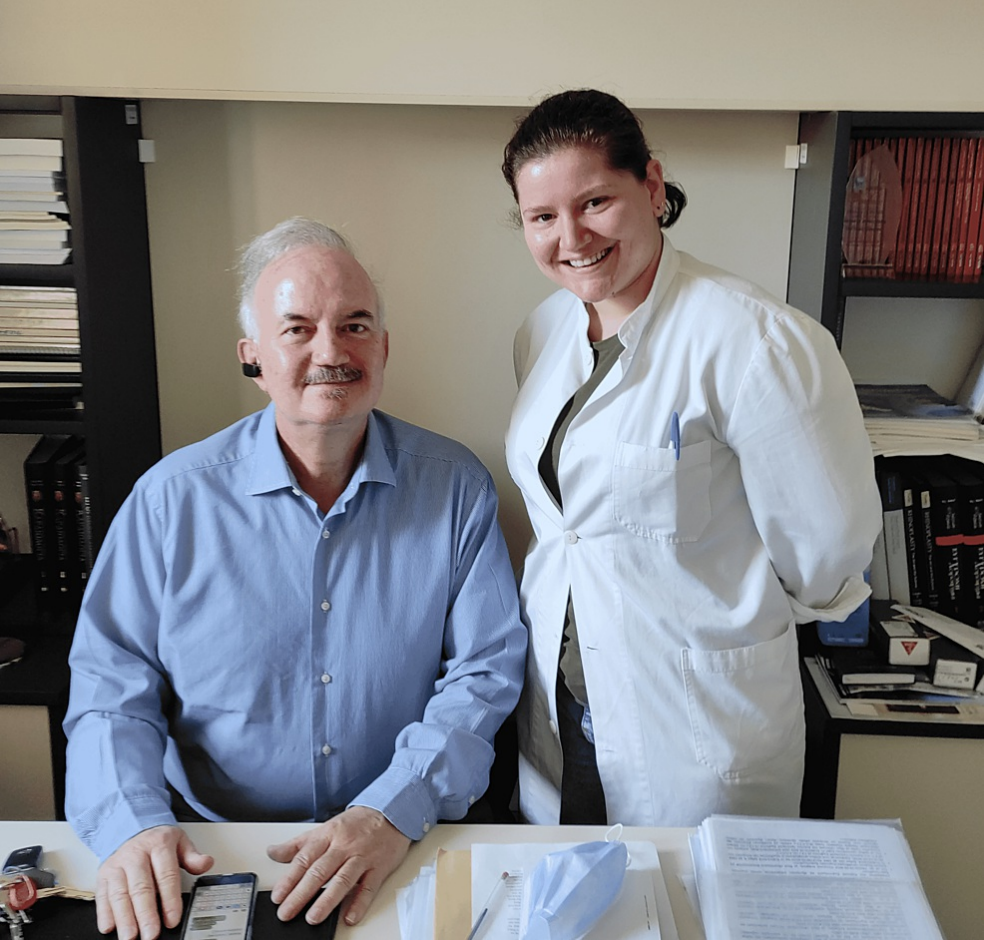
Me and my professor

Given the clinical and scientific evidence that cochlear implants minimize the effects of inadequate auditory influences and increase overall verbal development, they are highly recommended [6]. I am a firm believer in their use in children and, more broadly, in anyone who meets the criteria for implantation and wants to improve their quality of life. Deafness, from my perspective, is a disabling condition that must be remedied. Personally, I feel tremendously fortunate to have been given the freedom to pursue my aspirations and live the life I have always wished to live. Finally, it is worth noting that the surgeon who performed my cochlear implant surgery is now my professor and director of the clinic where I am pursuing my residency (Figures 1, 2).

## Conclusions

Cochlear implants are considered a viable therapeutic option for deaf children and adults who meet the implant criteria. Over the decades, we have witnessed passionate debates about communication philosophies related to deaf or hard-of-hearing children, the nature and concept of deafness, the value and diversity of deaf culture, the purpose of deaf education, and the best education system for deaf or hard-of-hearing children. There are two broad camps in the debate over cochlear implantation: those who embrace the technique and those who oppose it. In this article, I have described my own journey from total deafness to the beginning of a dream career as an otorhinolaryngology resident.

## References

[1] Mudry A, Mills M (2013). The early history of the cochlear implant: a retrospective. JAMA Otolaryngol Head Neck Surg.

[2] Krogmann RJ, Al Khalili Y (2022). Cochlear Implants. https://pubmed.ncbi.nlm.nih.gov/31335000/.

[3] Sampaio AL, Araújo MF, Oliveira CA (2011). New criteria of indication and selection of patients to cochlear implant. Int J Otolaryngol.

[4] Teagle HF (2012). Cochlear implantation for children: opening doors to opportunity. J Child Neurol.

[5] Nikolopoulos TP, Vlastarakos PV (2010). Treating options for deaf children. Early Hum Dev.

[6] Archbold S, Harris M, O'Donoghue G, Nikolopoulos T, White A, Richmond HL (2008). Reading abilities after cochlear implantation: the effect of age at implantation on outcomes at 5 and 7 years after implantation. Int J Pediatr Otorhinolaryngol.

[7] Okubo S, Takahashi M, Kai I (2008). How Japanese parents of deaf children arrive at decisions regarding pediatric cochlear implantation surgery: a qualitative study. Soc Sci Med.

[8] Nikolopoulos TP, Lloyd H, Archbold S, O'Donoghue GM (2001). Pediatric cochlear implantation: the parents' perspective. Arch Otolaryngol Head Neck Surg.

[9] Hyde M, Power D (2006). Some ethical dimensions of cochlear implantation for deaf children and their families. J Deaf Stud Deaf Educ.

[10] Pass L, Graber AD (2015). Informed consent, deaf culture, and cochlear implants. J Clin Ethics.

[11] Lee C (2012). Deafness and cochlear implants: a deaf scholar's perspective. J Child Neurol.

[12] Peters E (2000). Our decision on a cochlear implant. Am Ann Deaf.

[13] Cooper A (2019). Hear me out: hearing each other for the first time: the implications of cochlear implant activation. Mo Med.

[14] Crouch RA (1997). Letting the deaf be deaf. Reconsidering the use of cochlear implants in prelingually deaf children. Hastings Cent Rep.

